# True polyploid meiosis in the human male

**DOI:** 10.1590/1678-4685-GMB-2017-0219

**Published:** 2018-05-21

**Authors:** Peter L. Pearson, Kamlesh Madan

**Affiliations:** 1 Universidade de São Paulo Universidade de São Paulo Instituto de Biociências Departmento de Genética e Biologia Evolutiva SP Brazil Center for Human Genome and Stem Cell Research, Departmento de Genética e Biologia Evolutiva, Instituto de Biociências, Universidade de São Paulo, SP, Brazil; 2 Leiden University Medical Center Leiden University Medical Center Department of Clinical Genetics Leiden The Netherlands Cytogenetics Laboratory, Department of Clinical Genetics, Leiden University Medical Center, Leiden, The Netherlands

**Keywords:** Polyploidy, meiosis, human, male

## Abstract

Polyploidy does not usually occur in germinal cells of mammals and other higher vertebrates. We describe a unique example of mosaic autotetraploidy in the meiosis of a human male. Although the original observations were made in the late 1960s, we did not publish them at that time, because we expected to detect further examples that could be described together. However, this did not occur and we have now decided to make the observations available to demonstrate that polyploidy in mammalian male meiosis can arise at a higher frequency than expected by random polyploidization of individual meiotic cells, by either DNA duplication or cell fusion prior to synapsis. This is the first description of a population of primary spermatocytes exhibiting multivalent formation at leptotene /diakinesis in human spermatogenesis, with ring, chain, frying pan and other types of quadrivalents, typical of autotetraploidy. As many of the polyploid configurations showed apoptotic breakdown, it is likely that diploid and/or aneuploid spermatozoa would have rarely or never resulted from this mosaic autotetraploid meiosis.

There is only one published report describing pairing and meiotic crossing over by electron microscopic analysis of the synaptonemal complex of a single tetraploid primary spermatocyte during spermatogenesis in the mouse (Solari and Moses, 1977). Later, Codina-Pascual *et al.* (2006) also published a single tetraploid pachytene cell using SC analysis to show several quadrivalents exhibiting crossing over in a human male. As far as we are aware, these single cell observations are the only existing descriptions of true autotetraploid meiotic behavior in mammals and are referred to further below.

We wish to bring to your attention a unique example of mosaic autotetraploidy in human male meiosis based on observations made in the late 1960s at the MRC Population Genetics Unit in Oxford. We did not publish the results at the time in anticipation that other cases would be discovered that could be published together; regrettably, this did not occur. Now, ~ 50 years later, given the continuing uniqueness of the observations, we wish to make them available for the historical record whilst we still can, using the original chromosome photographs made at that time. These demonstrate that true polyploid meiosis, presumably resulting from polyploidy arising in spermatogonia or spermatocytes prior to synapsis (chromosome pairing), was detected in a mosaic, single human testicular biopsy, at an estimated frequency of ~3%.

The images we present in Figures 1-4 are from a testicular biopsy taken from a 75-year-old man of proven fertility, receiving surgical repair for a severe hernia. Examination of several hundred primary spermatocytes from air-dried preparations showed that ~3% of cells at diplotene/diakinesis had multivalent structures characteristic of autotetraploidy. Unfortunately, these observations were made before synaptonemalcomplex analysis (SC) or the ability to define centromere positions by deferential chromatin staining of AT rich DNA satellite fractions adjacent to centromeres by DAPI or Hoechst 33258, became available in the early 70s; these methods would have been perfect to confirm the multivalent features of true autotetraploidy in many of the primary spermatocytes encountered in our patient.

**Figure 1:**
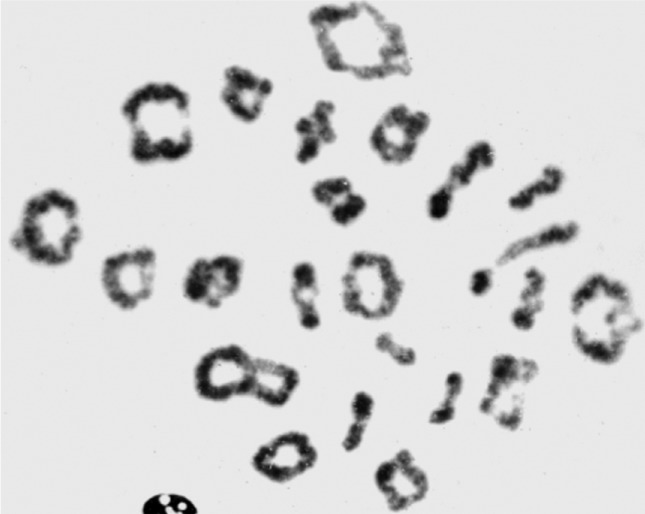
Diploid diakinesis with extremely low chiasma count of 38. The patient exhibited a much lower average chiasma count than usually observed in human males (range 46 – 49) with either diakinesis or SC analysis. Could this have resulted from his extremely advanced age? Several other studies in cohorts of younger males have not detected a significant age-related reduction in meiotic recombination frequency.

**Figure 2 f2:**
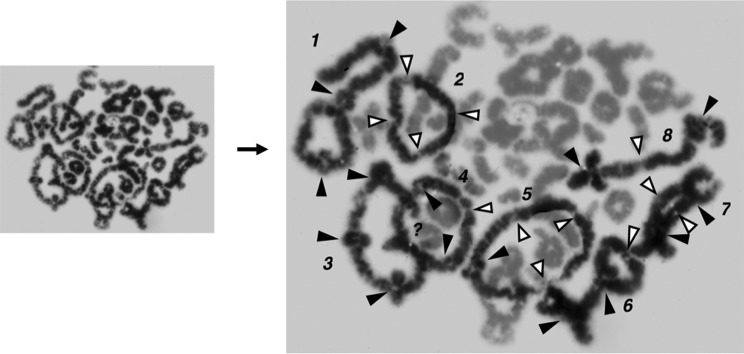
Contrast enhancement to distinguish selected multivalents within an auto-tetraploid diakinetic spermatocyte. Positions of incompletely terminalised chiasmata are indicated by black arrows and the presumed position of completely terminalised chiasmata, by white arrows. Quadrivalents 2, 3, 4 and 5 are rings, quadrivalent 8 is a chain, quadrivalent 1 has a classical frying pan structure and quadrivalents 6 and 7 are intermediates. Other quadrivalents are certainly present, but were either too small or lacked interstitial incompletely terminalised chiasmata to be so identified.

**Figure 3 f3:**
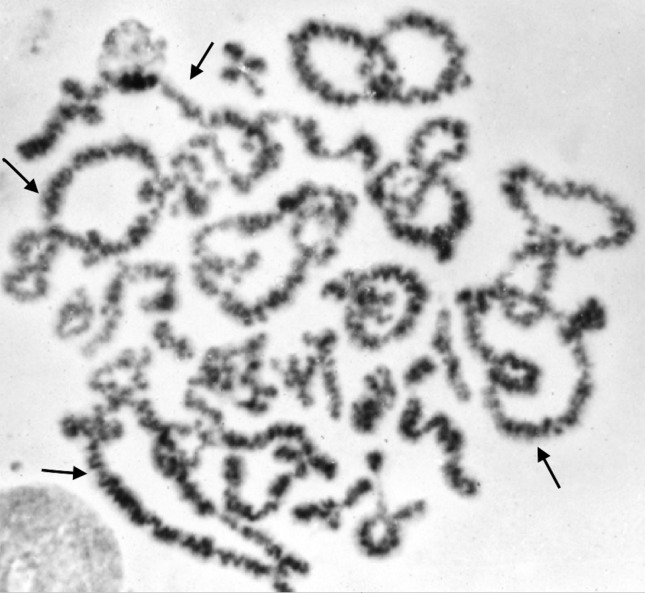
4n diplotene with arrowed quadrivalents.

**Figure 4:**
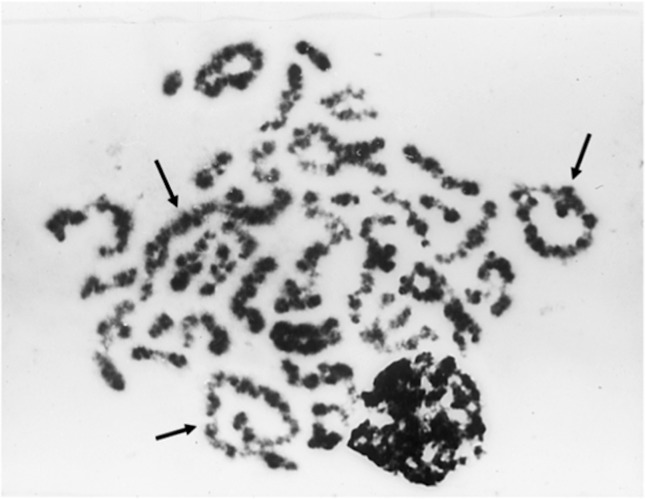
Apoptotic 4n diakinesis with arrowed quadrivalents.

Constitutional polyploidy is widely described in plants and in some invertebrate (e.g., flatworms, leeches, brine shrimps) and lower vertebrate species (salmonids and cryprinids), but is either extremely rare or non-existent in mammals. A single claim of putative allo-tetraploidy in the red vizcacha rat (Gallardo *et al.*, 1999; Gallardo *et al.*, 2006) was interpreted as diploidy despite the increased chromosome number (Svartman *et al.*, 2005). Meiotic diakinesis in many autopolyploid organisms, for example, *Chrysanthemum leucanthemum* (Ox-eyed daisy) shows the typical multivalent chromosome structures of autotetraploidy (Pearson, 1967), similar to those observed in our patient.

Polyploid spermatogonial cells have been described in the mouse (Fechheimer, 1961). Ford and Evans (1971) found ~ 10% polyploid MI cells in air-dried preparations from two mice with absence of multivalent structures, (which would be expected if true tetraploidy existed prior to pachytene). The authors concluded that the observed polyploid cells had arisen either by post zygotene cell fusion or resulted from juxtaposition of two cells during chromosome spreading by air-drying. Syncytia of adjacent spermatogonia in the rat (Moens and Hugenholtz, 1975) contain germ cells drawn into synchronous chromosome contraction and cell division, because of cytoplasmic connections between all spermatogonia and/or spermatocytes within a syncytium. This results in a uniform appearance of meiotic chromosomes in adjacent germ cells and would tend to keep the germ cells attached to each other during the air-drying procedure. Beatty *et al.* (1975) came to a similar conclusion as Ford and Evans (1971), since they found no diploid sperm in 14 strains of mice, although ~10% of primary spermatocytes were tetraploid. In 1977, Solari and Moses (1977) published clear electron microscopic images of the SC involving quadrivalents in a single mouse spermatocyte showing switching between multiple partners during synapsis. Codina-Pascual *et al.* (2006) found a single tetraploid pachytene cell in the testis of a normal man by SC analysis in which they identified pairing elements and position of crossing over in five quadrivalents.

Polyploid cells regularly occur in some highly differentiated tissues, such as hepatocytes, but true constitutive tetraploidy, although described in some human new-borns (Stefanova *et al.*, 2010), is extremely rare. Tetraploidy has been claimed to exist in human male meiotic cells: Skakkebaek *et al.* (1973) reported tetraploidy in spermatogonia as well as primary and secondary spermatocytes, in both fertile and infertile men (2.6% and 4.4%, respectively). However, no multivalents were observed and, as already stated, this observation probably resulted from adjacent synchronous diploid primary spermatocytes merging together during air-drying.

Apart from the single cell observation of Codina-Pascual *et al.* (2006), multivalent structures showing crossovers in tetraploid cells in human males have never been previously published. A noticeable number of primary spermatocytes in our patient were first viewed as large balls of entangled meiotic chromosomes, before it was realized that in fact they were polyploid primary spermatocytes that were not well spread: this inability to spread probably resulted from the 92 chromosomes lying within the cytoplasm of a single cell, and not in adjacent cells attached by cytoplasmic strands in a syncytium. Similar “autotetraploid” images were not observed in any of the many other human testicular biopsies (~70) we analyzed at the time. In retrospect, this visual difference supports our conclusion that our patient was unique in his tetraploid characteristics. Although auto-tetraploidy of the leptotene/diakinetic cells that we present in Figures 1 to 4, could not be confirmed by SC analysis or differential centromere staining, both techniques being published in the early 1970s following our observations, some diplotene and diakinetic cells clearly show multivalent structures typical of autotetraploidy, mainly evidenced by the increased size of some of the multivalents and the position and greater frequency of incompletely terminalised chiasmata than expected in bivalents. For example, in the tetraploid diakinesis depicted in Figure 2, the larger size of the identified quadrivalents was the primary selection parameter, secondarily supported by the number and position of incompletely terminalised chiasmata: the position of completely terminalised chiasmata can only be surmised. However, in multivalents where at least one incompletely terminalized chiasma is present, the position of completely terminalized chiasmata can be predicted much more accurately given the geometrical symmetry of the multivalents. Accordingly, based on the methods available to us in the late 1960s, our estimate of the number of multivalents present in individual primary spermatocytes was certainly too low. Further, the substantial level of tetraploid cells exhibiting multivalents in our patient suggests that his testis was a structural mosaic of autotetraploid and diploid spermatocytes, and not the result of an accumulation of occasional random cell fusions or DNA duplications.

It is possible that the patient’s advanced age contributed to the likelihood of structural testicular mosaicism: the human male germinal epithelium continues to divide and generate gametes throughout life with a well-documented paternal age-related increase in the frequency of sporadic point mutations at many loci (Kong *et al.*, 2012). Mutation at the *ADHD* gene that gives rise to achondroplasia is entirely paternal in origin and disease frequency at birth directly increases with paternal age. The usual presumption is that the large number of cell divisions that take place in the testis throughout life produces an increasing accumulation of many independent point mutations. However, the intriguing study of Dakouane-Giudicelli *et al.* (2008) examined the frequency of the G1138A mutation in both living semen donors (age range 30 – 65 years) and multiple testicular biopsies from post-mortem donors (age range 53 to 95 years). Although they found absence of an age-related increase in mutation frequency in semen samples possibly due to the lower age range of the sample, there was a clear and significant difference in mutation frequency observed between two biopsies of two of the donors. The authors conclude that such differences in “mutation concentration” arose in the testis through localized mosaicism. We think it likely that the same applies to our analysis, and that the observed 3% autotetraploidy is only relevant to mosaicism in the particular biopsy we studied and not to the entire gonad.

Given the significant number of multivalents with their predisposition to result in non-disjunction, aneuploidy could also be expected in addition to diploidy in individual spermatozoa. Since many of the polyploid spermatocytes exhibiting multivalents also showed signs of chromatin degeneration (now referred to as apoptosis), it is likely that the majority of multivalent bearing spermatocytes would have failed to complete meiosis and produced viable diploid sperm. Regrettably, we did not examine the DNA content of the patient’s sperm at the time, although the method was available to us, so were unable to conclude whether the observed mosaic tetraploid meiosis increased the proportion of either diploidy and/or aneuploidy in his spermatozoa.

Diploid sperm are found in ~ 0.1% (range 0.05% - 0.47%) of sperm in normal fertile males (Martin *et al.*, 1996; Guttenbach *et al.*, 1997; Vera *et al.*, 2012). Triploidy is one of the most frequent findings in human spontaneous abortions and occurs in 1-3% of all recognized pregnancies. The proportion of triploids with double contributions from either the father (diandric triploids) or the mother (digynic triploids) remains a matter of on-going controversy (Baumer *et al.*, 2000; Zaragosa *et al.*, 2000). In any case, it has been shown that while the majority of diandric triploids arise by dispermy, a significant proportion also result from fertilization by diploid sperm derived from errors at MI or MII (Zaragosa *et al.*, 2000). Egozcue *et al.* (2002) suggested that diandric triploids produced by normal males arise more frequently by dispermy, whereas those produced by oligospermic males derive from fertilization by diploid sperm.

In conclusion, although we have shown that mosaicism for gonadal autotetraploidy arose above the level of random polyploidisation of individual spermatocytes in the examined human testis, it is unlikely that a significant proportion of tetraploid germinal cells would have completed meiosis and produced diploid sperm, given that the chromatin degeneration (apoptosis) observed in many of the polyploid cells is one of the main barriers shown to prevent progression of abnormal progenitor cells through spermatogenesis (Moens and Hugenholtz, 1975)
